# Regulatory role of the lncRNAs MIAT and PVT1 in Behçet’s disease through targeting miR-93-5p and miR-124-3p

**DOI:** 10.1186/s10020-024-00914-8

**Published:** 2024-09-24

**Authors:** Asmaa A. ElMonier, Olfat G. Shaker, Shimaa O. Ali

**Affiliations:** 1 Department of Biochemistry, Faculty of Pharmacy, Cairo University, Cairo, Egypt; 2 Department of Medical Biochemistry and Molecular Biology, Faculty of Medicine, Cairo University, Cairo, Egypt

**Keywords:** MIAT, PVT1, miR-93-5p, miR-124-3p, SOD-2, MICA

## Abstract

**Background:**

Noncoding RNAs play pivotal roles in the process of autoimmune diseases. However, the definite contributions of these molecules to Behçet’s disease (BD) are still unknown. This study aimed to explore the clinical value of a novel competing endogenous (ce) RNA network in the pathogenesis of BD and to assess its use in primary diagnosis.

**Methods:**

Bioinformatic analysis was applied to construct a BD-related ceRNA network: lncRNA (MIAT and PVT1)-miRNA (miR-93-5p and miR-124-3p)-mRNA (SOD-2 and MICA). Blood was obtained from 70 BD patients and 30 healthy subjects, and the serum expression of the tested RNAs was estimated via quantitative real-time PCR (qPCR). Serum tumor necrosis factor-alpha (TNF-α) levels were also determined. The associations between these RNAs were further analyzed, and receiver operating characteristic (ROC) curve and logistic regression analyses were employed to validate their diagnostic and prognostic values.

**Results:**

The expression levels of the lncRNAs PVT1 and miR-93-5p were significantly increased, whereas those of the lncRNAs MIAT and miR-124-3p, as well as those of the SOD-2 and MICA mRNAs, were significantly decreased in BD patients compared with controls. BD patients had significantly higher serum TNF-α levels than controls did. ROC curve analysis indicated that the selected RNAs could be candidate diagnostic biomarkers for BD. Moreover, the highest diagnostic efficiency was achieved with the combination of MIAT and miR-93-5p or PVT1 and miR-124-3p with either SOD-2 or MICA. Logistic regression analysis revealed that all RNA expression levels could be predictors for BD.

**Conclusion:**

Mechanistically, our research revealed a novel ceRNA network that is significantly disrupted in BD. The findings reported herein, highlight the noncoding RNA-molecular pathways underlying BD and identify potential targets for therapeutic intervention. These insights will likely be applicable for developing new strategies for the early diagnosis, management and risk assessment of BD as well as the design of novel preventive measures.

*Trial registration* The protocol for the clinical studies was approved by Cairo University’s Faculty of Pharmacy’s Research Ethics Committee (approval number: BC 3590)

**Supplementary Information:**

The online version contains supplementary material available at 10.1186/s10020-024-00914-8.

## Introduction

Behçet’s disease (BD) is a rare inflammatory immune-related disorder with uneven remission and recurrence phases. BD is initially defined by numerous types of clinical involvement, including uveitis, skin abrasions, and genital and oral aphthous ulcers. However, there may also be other manifestations that impact more than one organ, leading to a constellation of clinical phenotypes with varying prognoses (Seyahi 2019; Uygunoglu and Siva 2021; Tugal-Tutkun 2023). These clinical signs can manifest separately or in combination in the same patient (Yazici et al. 2021). The mortality rate in BD patients is high and is predominantly associated with vascular and neurological phenotypes (Seyahi et al. 2023).

Owing to the complexity of the disease and its distinct geographic distribution, BD is thought to be influenced by both environmental and genetic factors (Mattioli et al. 2021; Zhang et al. 2023). BD diagnosis is primarily based on mucocutaneous symptoms due to the lack of widely accepted diagnostic laboratory tests for the disease. The most commonly used and widely documented diagnostic standard in this field is an oral ulcer combined with either of the following: typical cutaneous lesions, typical eye lesions, or a positive skin pathology test. These criteria were established by the International Study Group for Behçet’s Disease (ISBD) (1990).

Although its exact etiology has not yet been clarified, the intricate development of BD may involve genetic and epigenetic elements, such as correlations with human leukocyte antigen (HLA) and non-HLA genes, polymorphisms of microRNAs (miRNAs), immunological routes involving neutrophils, and many immune-mediated damage machinery (Mahmoudi et al. 2022).

Clinical studies have provided evidence of the potential resemblance of BD to spondyloarthropathies (SpA), establishing BD as a major histocompatibility complex (MHC)-I-opathy in humans. The associations of particular HLA class I sets, specifically HLA-B∗51 and HLA-B∗27, with the progression of BD and SpA, respectively, support this similarity (McGonagle et al. 2015; Gul 2015; Takeuchi et al. 2021).

In BD, miRNA dysregulation may lead to aberrant levels of immune-suppressive and inflammatory cells and cytokines. Despite this, much work needs to be done to completely comprehend the molecular pathways of miRNAs and BD, as the regulatory mechanisms of miRNAs-mRNAs (messenger RNAs) in BD are still unknown. In the peripheral blood of BD patients, the expression levels of the T-cell-related miRNAs miR-25, miR-106b, and miR-93 have been reported to be dramatically increased, whereas the expression levels of miR-146a and miR-155 are decreased (Ahmadi et al. 2019). In addition, miR-326, miR-155, and miR-23b have been demonstrated to participate in the regulation of BD through multiple signaling pathways, such as protein kinase B/mammalian target of rapamycin (Akt/mToR), Notch, and nuclear factor kappa B (NF-κB) (Gu et al. 2023).

Besides, hyperactivated neutrophils are key elements in the etiopathogenesis of BD, known as neutrophilic vasculitis. These granulocytes produce more superoxide and engage in phagocytosis, which may contribute to clot formation by oxidizing fibrinogen. Neutrophil activation promotes thrombosis formation in BD, suggesting a link between altered fibrinogen structure, neutrophil activation, and augmented reactive oxygen species (ROS) production (Batu 2020; Emmi et al. 2019). BD is strongly linked to proinflammatory cytokines such as interleukin (IL)-1, 10, 17, 21, and tumor necrosis factor-α (TNF-α) (Mohammed et al. 2022).

The majority of genetic variations associated with complex human illnesses, including autoimmune diseases, are found in noncoding parts of the genome, particularly long noncoding RNAs (lncRNAs) (Iyer et al. 2015). While research on lncRNAs is still in its early stages, growing evidence indicates their involvement in a variety of biological processes, such as the immune cell cycle, differentiation, and proliferation (Roy and Awasthi 2019). LncRNAs may contribute to disease progression through influencing gene expression indirectly by interacting with other regulatory molecules, such as miRNAs, or by directly binding to protein complexes, such as transcription factors (Johnson et al. 2024; Castellanos-Rubio and Ghosh 2022). LncRNAs also regulate the transcription and synthesis of inflammatory cytokines and genes involved in the immune response (Sigdel et al. 2015). The lncRNA myocardial infarction-associated transcript (MIAT) can serve as a sponge of miR-182-5p to further affect the NF-κB pathway and other biological behaviors (proliferation, colonizing, apoptosis, and inflammation) in diabetic nephropathy (Dong et al. 2021). Previously, the lncRNA plasmacytoma variant translocation 1 (PVT1) was reported to regulate NF-kB signaling by targeting miR-145-5p in rheumatoid arthritis (RA) (Tang et al. 2020). Additionally, the lncRNA PVT1/miR‐488‐3p has been connected to the emergence of inflammatory diseases, such as osteoarthritis, by promoting the apoptosis of normal chondrocytes (Ma et al. 2020).

Despite research progress in the knowledge of BD, its diagnosis is often challenging because of its nonspecific clinical symptoms and the absence of noninvasive laboratory biomarkers. Moreover, there are unmet needs in differential diagnostics, monitoring, prediction, and treatment personalization, which create difficulties in clinical practice, making BD a complex disorder linked to a higher risk of morbidity. This research gap limits our comprehension of disease pathogenesis. Therefore, in this study, we used bioinformatics to acquire a deeper understanding of the involvement of lncRNAs and miRNAs in BD risk. This is the first study to discuss the expression of the lncRNAs MIAT and PVT1/miR-93-5p and the miR-124-3p/SOD-2 and MICA axes in BD, providing a basis and research direction for future studies.

## Subjects and methods

### Study population

One hundred participants were gathered from the Department of Rheumatology at Kasr Al-Ainy Hospital in Cairo, Egypt; 70 had BD, and the remaining 30 were age-matched healthy controls. Before sample collection and use for research purposes, all the subjects provided written informed consent consistent with the ethical standards of the Helsinki Declaration, and the protocol was approved by Cairo University’s Faculty of Pharmacy’s Research Ethics Committee for experimental and clinical studies (approval number: BC 3590).

Patients with BD were diagnosed on the basis of the ISBD (1990). Patients present with a variety of disease symptoms, including neurological affections, skin lesions, eye lesions, and genital and oral ulcerations. Patients included those with stable disease with well-managed symptoms or those with active BD, who displayed at least one BD symptom despite treatment. Conversely, the controls did not have any family history of BD or other autoimmune disorders.

Participants were excluded from the study if they had any of the following: autoimmune disease, cancer, chronic infection, or a new infection within a month of enrollment. The participants also provided the following information: age, gender, clinical presentation, fundus and central nervous system examination, and disease activity, as assessed by Behçet’s Disease Current Activity Form (BDCAF) and Behçet’s Syndrome Activity Score (BSAS) (Yilmaz et al. 2013).

### Blood sampling and biochemical analysis

Intravenous blood samples (5 ml) were collected from all contributors and used to obtain serum, where blood was left for 30 min at room temperature to allow it to clot, after which it was centrifuged for 15 min at 3000×*g*. The separated sera were stored at − 20 °C till further analysis. The serum concentration of tumor necrosis factor-alpha (TNF-α) was then measured via an enzyme-linked immunosorbent assay (ELISA) kit (Raybiotech, USA, Cat. No. ELH-TNFα) utilizing ELISA reader (Tecan, A-5082) at a wavelength of 450 nm, in accordance with the manufacturer’s instructions.

### Bioinformatics and rationale of lncRNAs, miRNAs, and mRNAs selection

First, the pertinent protein-coding genes involved in the pathogenesis of BD were identified via the Gene Atlas (2023). Using DIANA tools (2023), miR-93-5p and miR-124-3p were carefully chosen because they are epigenetic regulators of the SOD-2 and MICA genes. By means of the Diana Tools database (2023), the MIAT and PVT1 lncRNAs were selected as competing endogenous RNAs (ceRNAs) for miR-93-5p and miR-124-3p.

### RNA extraction and reverse transcription

A miRNeasy extraction kit (Qiagen, USA, Cat. No. 217184) was used to extract total RNA from the serum as stated in the manufacturer’s protocol. The resulting RNA concentration and purity were then assessed via a NanoDrop 2000 (Thermo Fisher Scientific, USA) at 260/280 nm so that a ratio > 1.8 was indicative of an acceptable RNA yield and purity. Next, the RNA was reverse-transcribed with a cDNA reverse transcription kit (Applied Biosystems, USA; Cat. No. 4368814) following the supplier’s instructions. The reactions were incubated in a thermal cycler, which was programmed as follows: at 25 °C for 10 min, at 37 °C for 110 min, and then at 87 °C for 5 s. The resulting cDNA was then stored at − 20 °C for subsequent analysis.

### Quantitative real-time polymerase chain reaction (qPCR)

Using specific primers provided by Eurofins, Germany, and Maxima SYBR Green qPCR Master Mix (Thermo Fischer Scientific, USA, Cat. No. K0222) as directed by the manufacturer, quantitative real-time polymerase chain reaction (qPCR) was used to measure the expression levels of the selected RNAs. Table 1 shows the sequences of primers used in the PCR.

**Table 1:** Primers’ sequences used in qPCR reactions of the selected RNAs

RNA	Primer sequence
MIAT	Forward 5′-GCACCTTGAGTGAATGTCAAGGCAG-3′
	Reverse 5′-TGGCAGCATCCAGCCGACACACAGG-3′
PVT1	Forward 5′-TGG AATGTAAGACCCCGACTCT-3′
	Reverse 5′-GATGGC TGTATGTGCCAAGGT-3′
MiR-93-5p	Forward 5′-CGCAAAGTGCTGTTCGTGC-3′
	Reverse 5′-AGTGCAGGGTCCGAGGTATT-3′
MiR-124-3p	Forward 5′-TCTTTAAGGCACGCGGTG-3′
	Reverse 5′-TATGGTTTTGACGACTGTGTGAT-3′
SOD-2	Forward 5′-CTAACGGTGGTGGAGAACCC-3′
	Reverse 5′-TGAGCCTTGGACACCAACAG-3′
MICA	Forward 5′-CCTGCAGTGGCGCCTAAA-3′
	Reverse 5′-GGCTCTTATACCCCATGCACT-3′
GAPDH	Forward 5′-CCCTTCATTGACCTCAACTA-3′
	Reverse 5′-TGGAAGATGGTGATGGGATT-3′
U6	Forward 5′-CTCGCTTCGGCAGCACATA-3′
	Reverse 5′-CGCTTCACGAATTTGCGTG-3′

*GAPDH* glyceraldehyde 3-phosphate dehydrogenase, *MIAT* myocardial infarction associated transcript, *PVT1* plasmacytoma variant translocation 1, *SOD-2* superoxide dismutase-2, *MICA* MHC class I polypeptide-related sequence A

The Step One real-time PCR system was used to conduct the PCR reactions, and the cycling conditions were as follows: initial denaturation at 95 °C for 10 min, 40 cycles at 95 °C for 15 s, and 60 °C for 60 s. Melting curve analysis was employed to guarantee the specificity of the reactions. Next, the relative expression levels of the chosen RNAs in each sample were ascertained using the 2^−ΔΔCT^ method (Schmittgen and Livak 2008). For MIAT, PVT1, SOD-2, and MICA, glyceraldehyde 3-phosphate dehydrogenase (GAPDH) was utilized as a housekeeping reference gene, whereas U6 was used for miR-93-5p and miR-124-3p.

### Statistical analysis

The normality of the distribution of the data was evaluated via the Shapiro‒Wilk test. For two-group quantitative nonparametric data comparisons, the results were analyzed via the Mann‒Whitney U test. Potential linear relationships between the studied RNA expression levels were explored via Spearman’s rank correlation test. The diagnostic value of serum RNA levels was evaluated via a receiver operating characteristic (ROC) curve, which interprets data from BD patients as truly positive and data from healthy controls as truly negative. The sensitivity and specificity of the findings were estimated by determining the area under the ROC curve (AUC). The positivity rates for each RNA were then calculated via the chi-square (χ^2^) test. The predictive power of the selected RNAs for BD was assessed through univariate analysis with logistic regression. P was set at < 0.05. All the statistical analyses were performed using IBM SPSS (Statistical Package for the Social Science; IBM Corp, Armonk, NY, USA) version 26 and GraphPad Prism version 8.0.1 (GraphPad® Software, San Diego, California, USA).

## Results

### Demographic, clinical and biochemical assessment

No statistically significant difference in age was observed between BD patients and controls, with mean ages of 35.24 ± 0.91 years and 37.17 ± 0.91 years, respectively. Most of the subjects in both groups were males (88.6% in the BD group and 60% in the control group). The clinical features of BD patients according to the diagnostic criteria are summarized in Table 2. Importantly, BD patients had significantly higher serum levels of TNF-α than controls did, as shown in Table 2.

**Table 2:** Demographic, clinical and biochemical measurements of the studied participants

Clinical features	BD patients (n = 70)	Controls (n = 30)
Age (years)	35.24 ± 0.91	37.17 ± 0.91
Gender		
Male n (%)	62 (88.6%)	18 (60%)
Female n (%)	8 (11.4%)	12 (40%)
Headache	20 (28.6%)	–
Oral ulcer	34 (48.6%)	–
Genital ulcer	6 (8.6%)	–
Erythema	22 (31.4%)	–
Skin involvement	8 (11.4%)	–
Joint arthralgia	38 (54.3%)	–
Joint arthritis	4 (5.7%)	–
New eye involvement	46 (65.7%)	–
New active nervous system involvement	4 (5.7%)	–
New active major vessel involvement	2 (2.9%)	
BDCAF	7 (5–8)	–
BSAS score/100	23 (15–38)	
TNF-α (pg/ml)	31.53 ± 2.002****	6.23 ± 0.603

Data are expressed as n (%) or mean ± SE (standard error) or median (25th–75th percentile)

BDCAF (transformed index score on interval scale 0:20): Behçet’s disease current activity form; BSAS: Behçet’s syndrome activity score; TNF-α: tumor necrosis factor-alpha

Significantly different at ****P < 0.0001

### Relative expression of the studied RNAs in serum

**Fig. 1:**
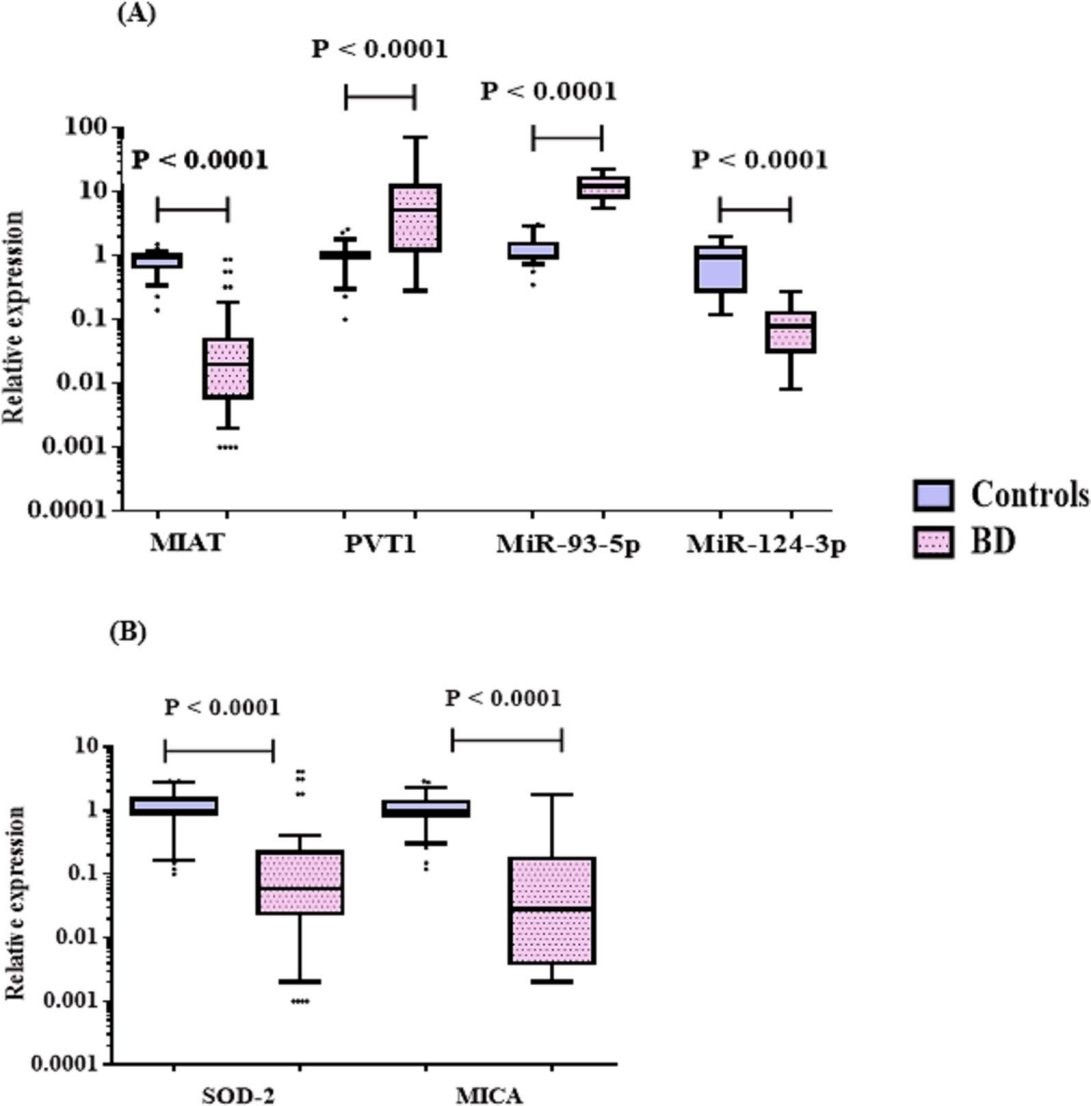
Relative expression levels of **A** lncRNAs (MIAT, PVT1) and miRNAs (miR-93-5p, miR-124-3p) and **B** target mRNAs (SOD-2 and MICA) in serum obtained from BD patients (n = 70) and controls (n = 30). The horizontal lines inside the box plots represent the median; the boxes mark the interval between the 25th and 75th percentiles. The whiskers denote the intervals between the 10th and 90th percentiles. The filled circles indicate data points outside the 10th and 90th percentiles. *MIAT* myocardial infarction-associated transcript, *PVT1* plasmacytoma variant translocation 1, *SOD-2* superoxide dismutase-2, *MICA* MHC class I polypeptide-related sequence A. Significant P values are indicated on the graph at P < 0.001

**Fig. 2:**
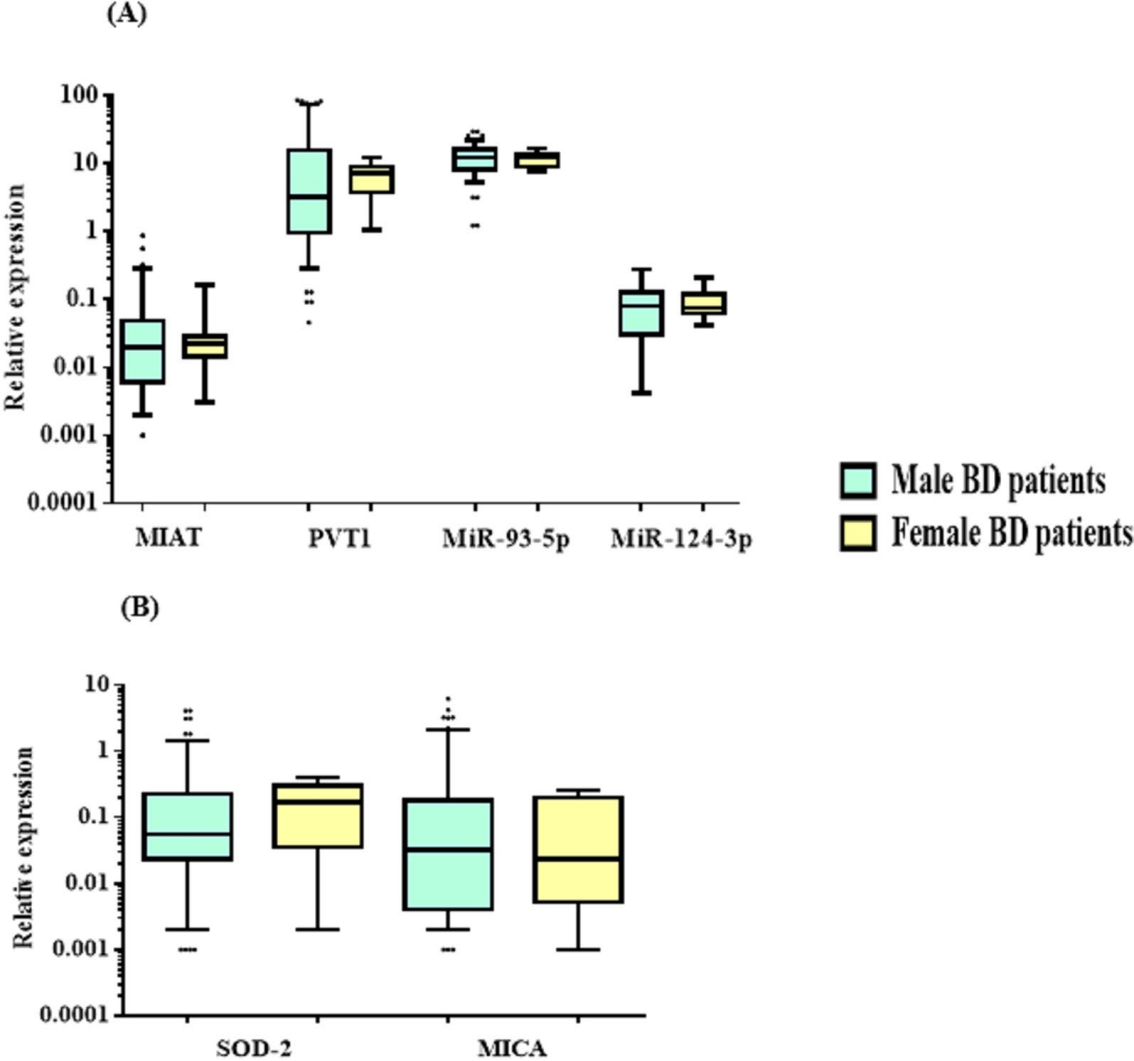
Relative expression levels of **A** lncRNAs (MIAT, PVT1) and miRNAs (miR-93-5p, miR-124-3p) and **B** target mRNAs (SOD-2 and MICA) in serum obtained from male BD patients (n = 62) and female BD patients (n = 8). The horizontal lines inside the box plots represent the median; the boxes mark the interval between the 25th and 75th percentiles. The whiskers denote the intervals between the 10th and 90th percentiles. The filled circles indicate data points outside the 10th and 90th percentiles. *MIAT* myocardial infarction-associated transcript, *PVT1* plasmacytoma variant translocation 1, *SOD-2* superoxide dismutase-2, *MICA* MHC class I polypeptide-related sequence A. No statistically significant differences were found between male and female BD patients

As shown in Fig. 1, BD patients presented significantly higher relative expression levels of the lncRNAs PVT1 and miR-93-5p in serum than healthy controls did, whereas the lncRNAs MIAT and miR-124-3p, as well as the mRNAs of SOD-2 and MICA, were expressed at lower levels. Notably, sex did not affect the relative expression levels of the studied RNAs, and no statistically significant differences were detected between male and female BD patients, as shown in Fig. 2.

### Correlation analysis

**Fig. 3:**
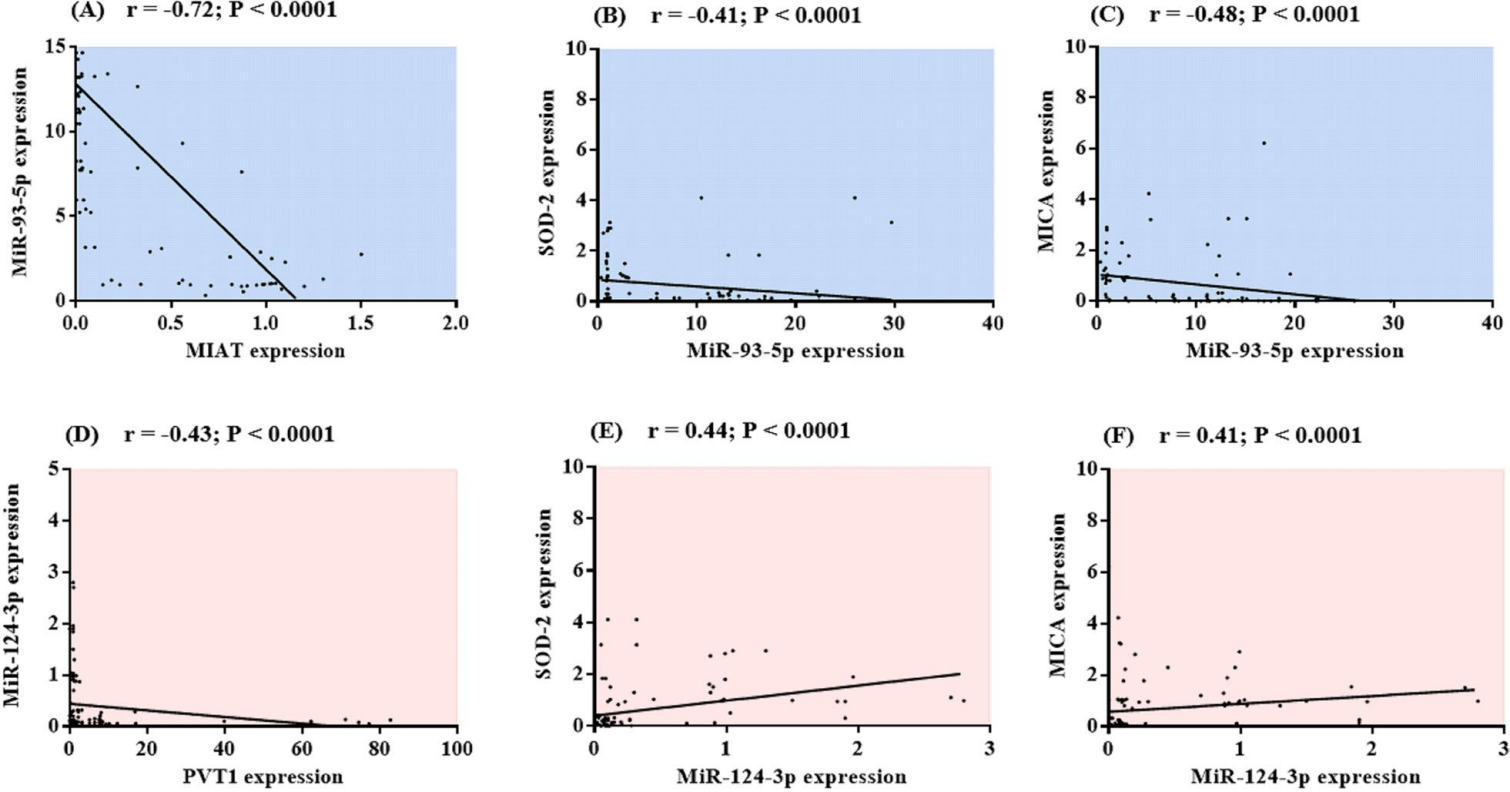
Spearman’s rank correlation analysis of **A** MIAT and miR-93-5p, **B** MiR-93-5p and SOD-2, **C** MiR-93-5p and MICA, **D** PVT1 and miR-124-3p, **E** MIR-124-3p and SOD-2, **F** MiR-124-3p and MICA in the studied participants. *r* correlation coefficient, *MIAT* myocardial infarction-associated transcript, *PVT1* plasmacytoma variant translocation 1, *SOD-2* superoxide dismutase-2, *MICA* MHC class I polypeptide-related sequence A. Significant P values are indicated on the graph at P < 0.001

The correlation analysis revealed that the MIAT expression level was negatively correlated with miR-93-5p; moreover, miR-93-5p was negatively correlated with SOD-2 and MICA, as depicted in Fig. 3A–C. Furthermore, PVT1 was negatively associated with miR-124-3p; however, miR-124-3p was positively correlated with SOD-2 and MICA, as depicted in Fig. 3D–F. These findings raised the possibility that high PVT1 expression may decrease the expression levels of SOD-2 and MICA by sponging miR-124-3p, whereas low MIAT expression may decrease the expression levels of these genes by increasing the expression of miR-93-5p, suggesting their involvement in the development of BD.

In addition, the serum TNF-α concentration was positively correlated with the expression levels of PVT1 and miR-93-5p. However, it was negatively correlated with MIAT, miR-124-3p, SOD-2, and MICA. The results of the correlation analysis between TNF-α and the studied RNAs are summarized in Supplementary Table 1.

### Diagnostic values and positivity rates of the relative expression levels of RNAs selected for BD

**Fig. 4:**
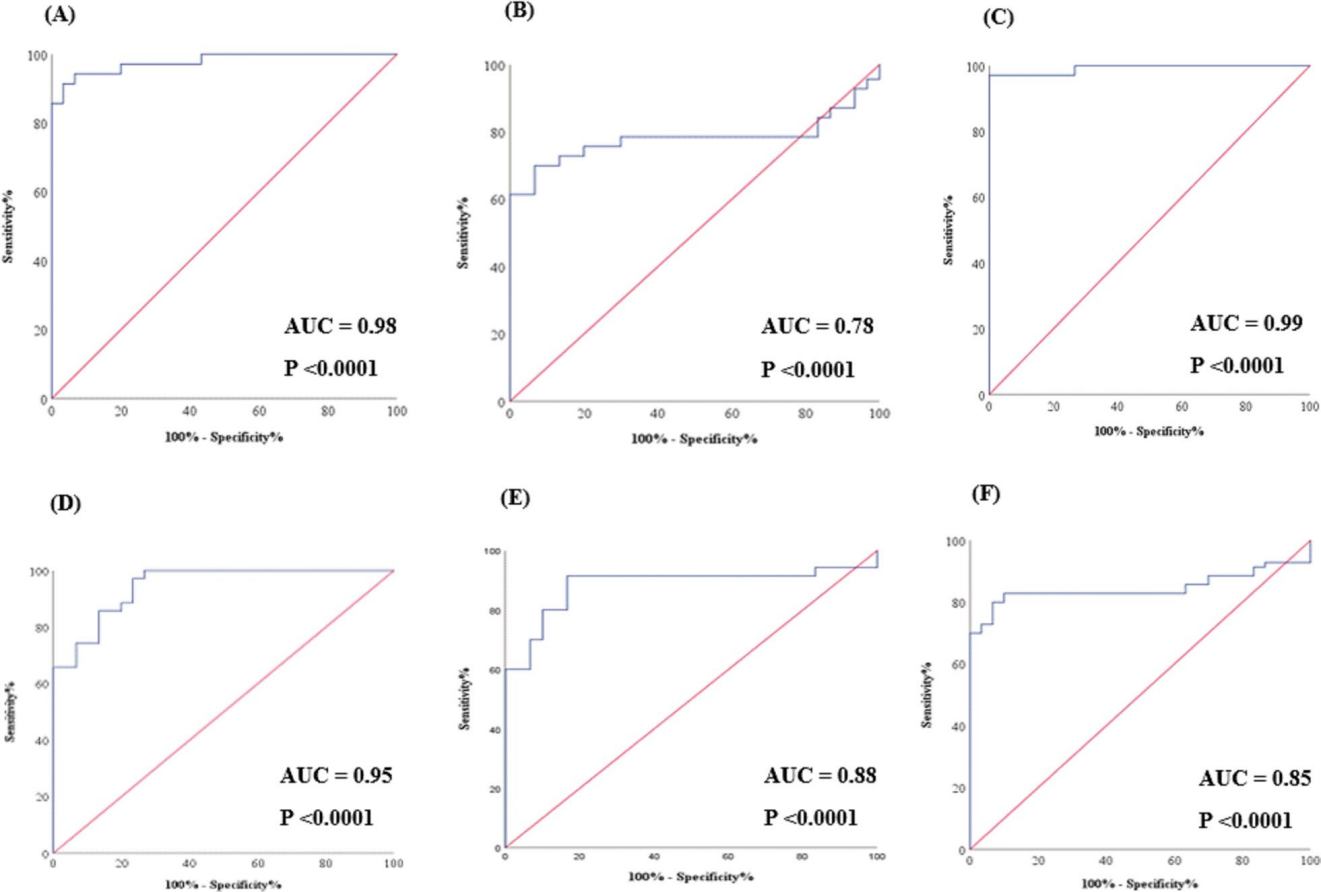
Receiver operating characteristic (ROC) curves of **A** MIAT, **B** PVT1, **C** MiR-93-5p, **D** MiR-124-3p, **E** SOD-2, and **F** MICA in the studied participants. *MIAT* myocardial infarction-associated transcript, *PVT1* plasmacytoma variant translocation 1, *SOD-2* superoxide dismutase-2, *MICA* MHC class I polypeptide-related sequence A. Significant P values are indicated on the graph at P < 0.001

The efficacy of the selected RNAs as BD serum diagnostic biomarkers was evaluated via ROC analysis, as shown in Table 3. The former analysis showed that all the selected RNAs can be applied in the differential diagnosis between BD patients and controls, where the diagnostic power of PVT1, SOD-2, and MICA relative expression levels was comparable, with AUCs of 0.78, 0.88, and 0.85, respectively. However, the diagnostic power of MIAT, miR-93-5p, and miR-124-3p was more potent, with AUCs of 0.98, 0.99, and 0.95, respectively, as depicted in Fig. 4A–F.

**Table 3:** ROC analysis of the selected RNAs in the studied participants

RNA	AUC	P-value	Cut-off value	Sensitivity (%)	Specificity (%)	PPV (%)	NPV (%)
MIAT	0.98	< 0.0001****	0.21	91.4	96.7	98.5	82.9
PVT1	0.78	< 0.0001****	1.82	70	93.3	96.1	57.1
miR-93-5p	0.99	< 0.0001****	3.14	97.1	100	100	93.8
miR-124-3p	0.95	< 0.0001****	0.29	97.1	76.7	90.7	92
SOD-2	0.88	< 0.0001****	0.46	91.4	83.3	92.8	80.6
MICA	0.85	< 0.0001****	0.26	80	93.3	96.6	66.7
Combination of MIAT, miR-93-5p and SOD-2	0.996	< 0.0001****	–	97.1	100	100	93.8
Combination of MIAT, miR-93-5p and MICA	0.998	< 0.0001****	–	98.6	100	100	96.7
Combination of PVT1, miR-124-3p and SOD-2	0.97	< 0.0001****	–	84.3	96.7	98.3	72.5
Combination of PVT1, miR-124-3p and MICA	0.97	< 0.0001****		100	83.3	93.3	100

*AUC* area under the curve, *PPV* positive predictive value, *NPV* negative predictive value, *MIAT* myocardial infarction associated transcript, *PVT1* plasmacytoma variant translocation 1, *SOD-2* superoxide dismutase-2, *MICA* MHC class I polypeptide-related sequence A

Significantly different at ****P < 0.0001

**Fig. 5:**
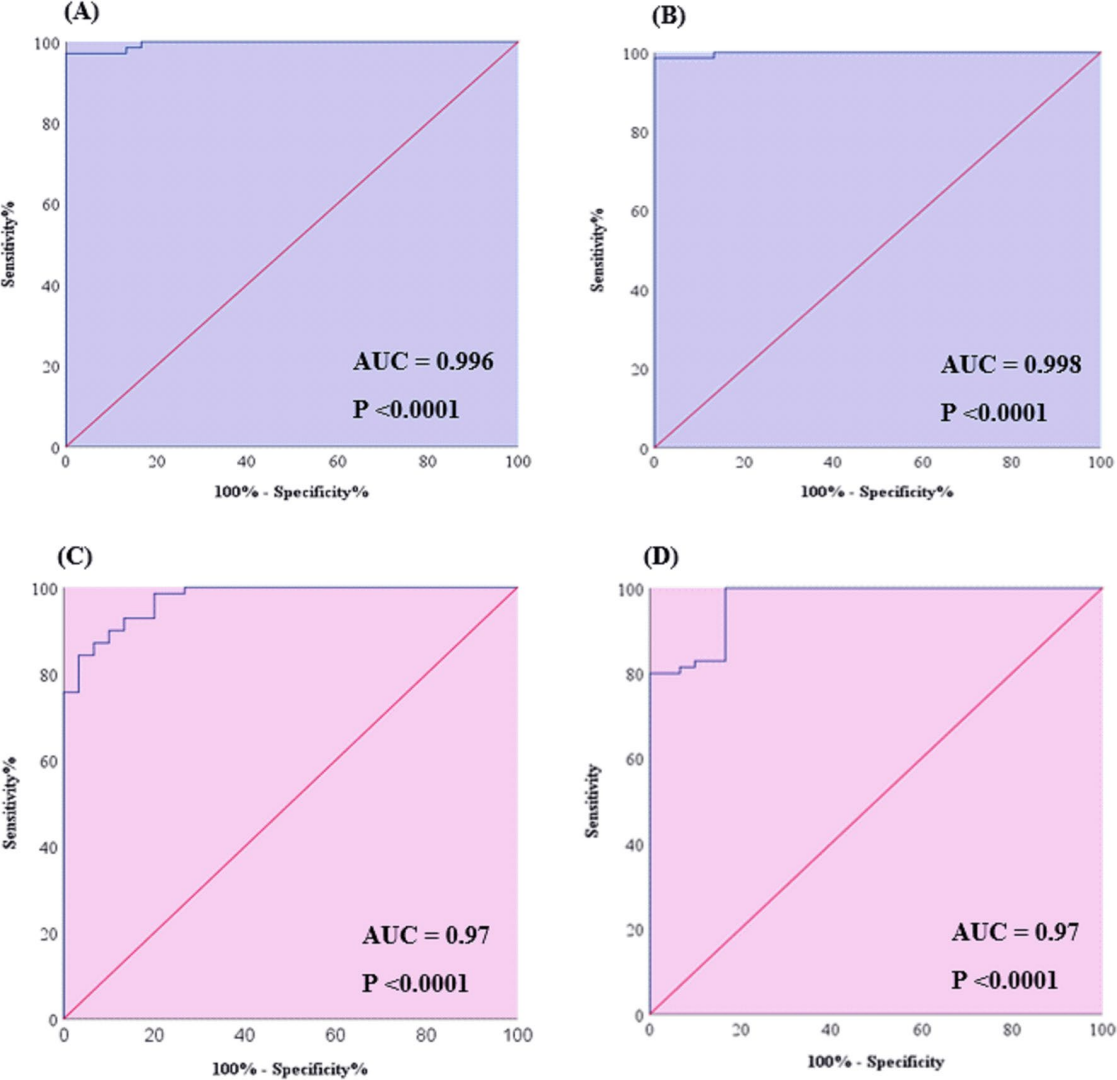
Receiver operating characteristic (ROC) curves of **A** combinations of MIAT, miR-93-5p and SOD-2. **B** Combinations of MIAT, miR-93-5p and MICA. **C** Combinations of PVT1, miR-124-3p and SOD-2. **D** Combinations of PVT1, miR-124-3p and MICA in the studied participants. *MIAT* myocardial infarction-associated transcript, *PVT1* plasmacytoma variant translocation 1, *SOD-2* superoxide dismutase-2, *MICA* MHC class I polypeptide-related sequence A. Significant P values are indicated on the graph at P < 0.001

Remarkably, the highest potential efficiency was found upon the combination of MIAT and miR-93-5p or PVT1 and miR-124-3p with either SOD-2 or MICA, where the AUC values for the MIAT combinations were 0.996 and 0.998, respectively, as shown in Fig. 5A, B, while those of the PVT1 combinations were 0.97 for both SOD-2 and MICA, as represented in Fig. 5C, D.

Each of the tested RNA positivity rates was determined by utilizing the calculated cutoff values for the recognition of BD, as outlined in Table 4. In BD patients, miR-93-5p and miR-124-3p had the maximum positivity rates, reaching 97.1% for both, followed by MIAT and SOD-2, with positivity rates of 91.4% for both; then, MICA and PVT1 had positivity rates of 80% and 70%, respectively.

**Table 4:** Positivity rates of the studied RNAs across the investigated groups

RNA	BD patients	Controls	X^2^	P-value
MIAT				
No. of +ve cases **(≤ 0.21)**	64 (91.4%)	1 (3.3%)	71.64	< 0.0001****
No. of −ve cases **(> 0.21)**	6 (8.6%)	29 (96.7%)		
PVT1				
No. of +ve cases **(≥ 1.82)**	49 (70%)	2 (6.7%)	33.71	< 0.0001****
No. of −ve cases **(< 1.82)**	21 (30%)	28 (93.3%)		
MiR-93-5p				
No. of +ve cases **(≥ 3.14)**	68 (97.1%)	0 (0%)	91.07	< 0.0001****
No. of −ve cases **(< 3.14)**	2 (2.9%)	30 (100%)		
MiR-124-3p				
No. of +ve cases **(≤ 0.29)**	68 (97.1%)	7 (23.3%)	61.02	< 0.0001****
No. of −ve cases **(> 0.29)**	2 (2.9%)	23 (76.7%)		
SOD-2				
No. of +ve cases **(≤ 0.46)**	64 (91.4%)	5 (16.7%)	54.87	< 0.0001****
No. of −ve cases **(> 0.46)**	6 (8.6%)	25 (83.3%)		
MICA				
No. of +ve cases **(≤ 0.26)**	56 (80%)	2 (6.7%)	46.36	< 0.0001****
No. of −ve cases **(> 0.26)**	14 (20%)	28 (93.3%)		

Significant differences were detected between the investigated groups using chi-square (χ^2^) test, utilizing the calculated cutoff values for the recognition of BD, as indicated by the bold values between brackets

MIAT: myocardial infarction associated transcript; PVT1: plasmacytoma variant translocation 1; SOD-2: superoxide dismutase-2; MICA: MHC class I polypeptide-related sequence A; +ve: positive; −ve: negative

Significantly different at ****P < 0.0001

The data of the ROC curve analysis and positivity rates of the studied RNAs suggested that all of them may be promising diagnostic markers of BD, with the highest efficiency found upon the combination of MIAT and miR-93-5p or PVT1 and miR-124-3p with either the SOD-2 or MICA genes, highlighting the possibility of a multimarker diagnostic approach for improved accuracy.

### Univariate logistic regression analysis

To assess the ability of the examined RNAs to predict BD, univariate logistic regression analysis was performed. The data in Table 5 illustrate that all the selected RNAs were verified as substantial predictors of BD; thus, these results may provide further competency for their suitable usage as reliable biomarkers for the prediction of BD.

**Table 5:** Univariate regression analysis for the predictive power of the studied RNAs with Behçet’s disease

RNA	Coefficient (β)	SE	P-value	Odds ratio	95% CI
Univariate analysis					
MIAT	− 7.12	1.32	< 0.0001****	0.001	0.00006–0.01
PVT1	0.63	0.22	0.005**	1.87	1.21–2.88
MiR-93-5p	1.37	0.43	0.001**	3.94	1.7–9.13
MiR-124-3p	− 10.104	3.09	0.001**	0.00004	< 0.0001–0.02
SOD-2	− 1.03	0.29	0.0004***	0.36	0.201–0.63
MICA	− 0.61	0.23	0.008**	0.54	0.35–0.86

P-values, OR (odds ratio) and 95% CI (confidence intervals) were estimated by using binary logistic regression model

*MIAT* myocardial infarction associated transcript, *PVT1* plasmacytoma variant translocation 1, *SOD-2* superoxide dismutase-2, *MICA* MHC class I polypeptide-related sequence A

Significantly different at **P < 0.01, ***P < 0.001 and ****P < 0.0001

## Discussion

The cause of BD is not fully understood, but genetic factors and environmental triggers may contribute to its development (Jo et al. 2022). Previous genetic investigations of BD have identified several strong genetic susceptibility loci for this illness (Ortiz-Fernández and Sawalha 2021). The purpose of this work was to systematically explain a novel network for the involvement of [lncRNA (MIAT and PVT1)-miRNA (miR-93-5p and miR-124-3p)-mRNA (SOD-2 and MICA)] and their connections to the downstream cytokine TNF-α in BD development in Egyptian patients. These molecules collectively form a ceRNA network through interactions among the lncRNAs, miRNAs, and mRNAs of BD target genes. Our research is the first to provide evidence of its association with susceptibility to BD.

In the present study, BD patients presented significantly increased expression levels of the lncRNA PVT1 and miR-93-5p, with significantly decreased expression levels of the lncRNA MIAT and miR-124-3p as well as the mRNAs of SOD-2 and MICA in the serum. An imbalance of lncRNAs plays a critical role in various complex conditions, such as cancer, as well as inflammatory and autoimmune diseases. LncRNAs identified in genome-wide association studies could influence the expression of specific disease-related genes, particularly through regulatory variations that might be responsible for an individual’s susceptibility to common diseases (Zhang et al. 2023).

The MIAT gene is located on human chromosome 12q12.1. The general consensus is that MIAT regulates genes both transcriptionally and posttranscriptionally. At the transcriptional level, MIAT interacts with nuclear factors in the nucleus to play a regulatory role; at the posttranscriptional level, it acts through ceRNA in the cytoplasm. Therefore, MIAT has the capacity to significantly affect important cellular processes, such as migration, cycle progression, apoptosis, and cell proliferation (Lu and Lu 2023; Da et al. 2020). This study demonstrated that MIAT levels were significantly lower in BD patients than in controls. Our findings contrast with those of a study on BD patients with noninfectious uveitis, where the lncRNA MIAT was significantly upregulated in their peripheral blood (Lu and Lu 2023).

MIAT, as an autoimmune disease-related gene, has a particular function in immune cells (Roy and Awasthi 2019). MIAT enhances systemic lupus erythematosus (SLE) development by interacting with miR-222 (Zhang et al. 2021). Additionally, a previous study revealed that MIAT downregulates IL-1β and TNF-α, suppressing macrophage inflammation (Wang et al. 2021).

The PVT1 gene is located on chromosome 8q24.21 (Ghetti et al. 2020). Studies have shown that the lncRNA PVT1 is related to the development of many inflammatory diseases (Ma et al. 2020). With respect to PVT1, BD patients had a noticeably greater fold change. In contrast, the expression of PVT1 was reported to be lower in the peripheral blood mononuclear cells of patients with relapsing–remitting multiple sclerosis than in those of healthy subjects (Eftekharian et al. 2017).

LncRNAs have well-studied regulatory mechanisms, including acting as ceRNAs to sponge complementary miRNAs (Zhang et al. 2023; Du et al. 2016). MIAT adjusts miR-411-5p by acting as a ceRNA that binds to miR-411-5p, thus hindering its action in hepatocellular carcinoma, providing evidence in favor of the role of lncRNA-mediated ceRNAs (Elmasri et al. 2024). In addition, sponging miR-3085-5p by MIAT has been demonstrated to at least partially mediate the activation of hepatic stellate cells (Zhan et al. 2023). Additionally, PVT1 has been reported to control breast cancer-related gene expression by competitively binding to miR-145-5p (Qu et al. 2023). Other studies have verified the role of PVT1/miR-486-5p in colorectal cancer (Khalafizadeh et al. 2024). Herein, bioinformatics analysis and clinical validation revealed that MIAT and PVT1 are direct targets of miR-93-5p and miR-124-3p, respectively. Correlation analysis in this study revealed that the expression levels of MIAT and PVT1 were negatively correlated with those of miR-93-5p and miR-124-3p, respectively.

The importance of miRNAs in innate and adaptive immunity, as well as their involvement in immune cell differentiation, has been demonstrated previously. MiRNAs are closely associated with the functioning of immune cells and the status of immune-related disorders. Consequently, the detection of circulating miRNAs serves as a valuable tool for diagnosing immune conditions (Yan et al. 2022). Previously, miRNA‒mRNA interaction analysis was conducted on autoimmune-dysregulated miRNAs to identify new targets for miRNAs that may be involved in the regulation of T-helper 17 (Th17) differentiation. In the former analysis, several miRNAs (miR-20b, miR-93, miR-20a, miR-152, miR-21, and miR-106a) were identified as Th17 differentiation inhibitors through interactions with positive effectors of this pathway (Honardoost et al. 2015). Moreover, miR-93-5p, miR-29a-3p, and miR-24-3p have been reported to be correlated with T-cell proliferation, differentiation, and the immune response (Zhu et al. 2019a).

Our patients presented higher expression values of serum miR-93-5p and lower miR-124-3p levels than normal controls did, suggesting their contribution to the development of BD. There are no existing reports on the application of miR-93-5p and miR-124-3p as diagnostic markers in BD. However, miR-93-5p has been documented as a potential therapeutic target, specifically in rheumatoid arthritis (RA) and multiple sclerosis (Zhu et al. 2019a). Additionally, circulating miR-124-3p has been suggested as a promising biomarker for the diagnosis of SLE, and it is also involved in the etiology of RA (Yan et al. 2022; Nakamachi et al. 2023).

MiRNAs regulate target mRNAs by combining with their 3’-untranslated region, affecting their translation (Yan et al. 2022). We predicted from DIANA tools that miR-93-5p and miR-124-3p could target SOD-2 and MICA, so we speculated that these miRNAs may affect the occurrence and development of BD by altering the transcription of SOD-2 and MICA. In accordance with our results, very low MICA concentrations were measured in the sera of BD patients in a prior study, but they were not significantly different from those of healthy individuals (Hervier et al. 2021). Contrary to our findings, in a former study, the MICA*049 allele was reported to be meaningfully more common in BD patients than in healthy controls in a Chinese cohort (Zhu et al. 2019b).

The MICA gene is positioned at the 46 kb centromeric region of HLA-B and functions as an adaptive and innate immunity gene. MICA acts as a ligand for natural killer group 2 member D receptors, which are expressed on natural killer cells (Baranwal and Mehra 2017). Studies have revealed that the MICA^*^009, MICA*019, and MICA*A6 alleles are associated with BD vulnerability (Lin et al. 2023; Khoshbakht et al. 2023). Importantly, the HLA-B/MICA genetic region is important not only in the overall pathogenesis of BD but also in ocular involvement (Casares-Marfil et al. 2023).

On the other hand, non-HLA regions linked to BD disease have also been identified by genome-wide association studies, along with polymorphisms in certain genes, including those encoding the intercellular adhesion molecule-1, TNF, endothelial nitric oxide synthase, vascular endothelial growth factor, manganese superoxide dismutase (MnSOD), endoplasmic reticulum aminopeptidase 1, cytochrome P450, IL-10, and IL-23 receptor genes (Al-Musawi et al. 2020; Nguyen et al. 2021). Previously, the MnSOD gene was significantly associated with BD in a group of Turkish patients (Uz et al. 2016). It has been shown that there is a correlation between MnSOD and autoinflammatory diseases, yet the exact mechanisms involved are still mostly uncertain (Liu et al. 2022). In addition, the presence of oxidative stress has been demonstrated in BD, but the activity of antioxidant enzymes, particularly SOD, is impaired and negatively associated with disease duration and activity (Harzallah et al. 2008). However, no study has yet linked the relationship between the SOD-2 gene and BD pathophysiology. In the present study, it has been speculated that both SOD-2 and MICA gene expression may significantly decrease through alterations in miR-93-5p and miR-124-3p expression levels.

Interestingly, our results demonstrated that the serum TNF-α level was greater in patients with BD than in controls and was negatively correlated with SOD-2. According to earlier evidence, TNF-α has been linked to the immune pathogenesis of BD. Oxidative stress is also known to increase TNF-α production (Akhter et al. 2023). The selected RNA data from the ROC curve analysis and positivity rates suggest that each of them could be used for diagnosing BD. The peak efficiency was attained upon combination of MIAT and miR-93-5p or PVT1 and miR-124-3p with either the SOD-2 or MICA genes. Logistic regression analysis indicated that all RNAs were statistically significant, suggesting that these RNAs might serve as easily detectable biomarkers in laboratory tests for predicting BD.

## Conclusion and future directions

Our study’s most significant achievement is the introduction of a novel multilevel gene monitoring network that is created on the basis of the relationships among lncRNAs, miRNAs, and mRNAs connected to the pathophysiology of BD. The ceRNA network data suggested that PVT1 overexpression might decrease the expression of SOD-2 and MICA by sponging miR-124-3p, whereas low MIAT expression may decrease the expression of the aforementioned genes by increasing the expression of miR-93-5p, indicating their participation in BD development and progression. The determination of such a combination has high potential for clinical application. Indeed, elucidating the intricate molecular pathways underlying BD, as provided by this study, could lead to the identification of clinically useful biomarkers for early diagnosis, the prediction of disease course, the selection of targeted therapies, and the optimization of patient outcomes, especially in regions with a high prevalence of BD. In addition, better biomarkers could facilitate personalized treatment tailored to disease endotypes and decrease delays in starting appropriate therapy. Nevertheless, efficient analysis of these ceRNA networks in BD needs more investigation into their diagnostic and prognostic utility. Incipient options such as serum lncRNAs and miRNAs are promising but warrant authentication in diverse ethnic populations. Moreover, the study involved a small number of cases, which limits its ability to fully represent the biochemical and cellular characteristics of all patients. Therefore, more cases are necessary to confirm these findings. Finally, coordinated efforts and substantial research, ranging from fundamental scientific discoveries to clinical interventions and beyond, are necessary to achieve the goal of discovering effective treatments for BD.

## Supplementary Information

Supplementary Material 1.

## Data Availability

The datasets used and/or analyzed during the current study are available from the corresponding author on reasonable request.

## Abbreviations

AUC: Area under the curve
BD: Behçet’s disease
BDCAF: Behçet’s Disease Current Activity Form
BSAS: Behçet’s Syndrome Activity Score
χ^2^: Chi-square
ceRNA: Competing endogenous RNA
ELISA: Enzyme-linked immunosorbent assay
GAPDH: Glyceraldehyde 3-phosphate dehydrogenase
HLA: Human leukocyte antigen
ISBD: International Study Group Criteria for Behçet’s Disease
lncRNA: Long noncoding RNA
MHC: Major histocompatibility complex
MIAT: Myocardial infarction-associated transcript
MICA: MHC class I polypeptide-related sequence A
miRNA: MicroRNA
MnSOD: Manganese superoxide dismutase
mRNA: Messenger RNA
NF-κB: Nuclear factor kappa B
NKG2D: Natural killer group 2 member D
PVT1: Plasmacytoma variant translocation 1
qPCR: Quantitative real-time polymerase chain reaction
r: Correlation coefficient
RA: Rheumatoid arthritis
ROC: Receiver operating characteristic
ROS: Reactive oxygen species
SLE: Systemic lupus erythematosus
SOD-2: Superoxide dismutase-2
SpA: Spondyloarthropathies (SpA)
Th17: T-helper 17
TNF-α: Tumor necrosis factor-α
