# Supplementary material for: Regulatory role of the lncRNAs MIAT and PVT1 in Behçet’s disease through targeting miR-93-5p and miR-124-3p
Source: Mol Med. 2024 Sep 24;30:157. doi: 10.1186/s10020-024-00914-8 (PMC11423507; doi:10.1186/s10020-024-00914-8)
Supplement: Supplementary file 1 — Supplementary Material 1. [file 10020_2024_914_MOESM1_ESM.docx]

**Supplementary table 1: Spearman's rank correlation analysis of TNF-α and the studied RNAs**

| **RNA** | **TNF-α** | **P-value** |
| --- | --- | --- |
|  | **r** |  |
| **MIAT** | -0.49 | < 0.0001 |
| **PVT1** | 0.24 | 0.02 |
| **MiR-93-5p** | 0.52 | < 0.0001 |
| **MiR-124-3p** | -0.52 | < 0.0001 |
| **SOD-2** | -0.47 | < 0.0001 |
| **MICA** | -0.3 | 0.003 |

r: correlation coefficient; MIAT: myocardial infarction associated transcript; PVT1: plasmacytoma variant translocation 1; SOD-2: superoxide dismutase-2; MICA: MHC class I polypeptide-related sequence A. Significant P-values are indicated in the table.
